# Supplementary material for: High red blood cell distribution width attenuates the effectiveness of Immune checkpoint inhibitor therapy: An exploratory study using a clinical data warehouse
Source: PLoS One. 2024 Aug 1;19(8):e0299760. doi: 10.1371/journal.pone.0299760 (PMC11293676; doi:10.1371/journal.pone.0299760)
Supplement: Attachment — Submitted filename: Response to Reviewers.docx [file pone.0299760.s004.docx]

Response to Reviewers

Manuscript ID: PONE-D-24-05510

Title: High Red Blood Cell Distribution Width Attenuates the Effectiveness of Immune Checkpoint Inhibitor Therapy: An Exploratory Study Using a Clinical Data Warehouse

Dear Dr. Patnaik,

We sincerely appreciate the time and effort that you and the reviewers have invested in evaluating our manuscript. We have carefully considered all comments and suggestions and have made the necessary revisions to address each point. Our detailed responses to the reviewers' comments are provided below. We believe these revisions have significantly improved the quality and clarity of our manuscript.

Reviewer #1 Comments:

Short title:

Comment: I think it is better to use "effectiveness" than "efficacy".

Response: The short title has been revised to "High Red Blood Cell Distribution Width Attenuates the Effectiveness of Immune Checkpoint Inhibitor Therapy".

Comment: It should sound better as "This study utilizes a Clinical Data Warehouse (CDW) to explore the prognostic significance of novel blood-based factors such as the neutrophil-to-lymphocyte ratio and red cell distribution width (RDW) to enhance the prediction of ICI therapy benefit."

Response: The background section of the abstract has been revised accordingly.

Comment: It misses the extended form of "NSCLC" and "EMR".

Response: The full forms "Non-Small Cell Lung Cancer (NSCLC)" and "Electronic Medical Record (EMR)" have been included at their first mention.

Abstract, Methods:

Comment: You should mention ROC curve analysis.

Response: ROC curve analysis has been mentioned in the abstract methods section. The sentence has been revised to "Logistic regression identified factors predicting ≤2 or ≥5 pembrolizumab doses as proxies for progression-free survival (PFS), and Receiver Operating Characteristic analysis was used to examine their predictive ability."

Abstract, Results:

Comment: You should mention the total number of patients or the number of patients in each cohort.

Response: The total number of patients and the number of patients in each cohort have been added to the abstract results section. The following sentence has been added; “A total of 609 cases (428 in the exploratory cohort and 181 in the validation cohort) from CDW and 44 cases from EMR were selected for study.”

Comments:

Line 51: It is better to use "suggests" than "affirms".

Line 52: It is better to use "initiation" than "commencement".

Response: The conclusions in the abstract have been revised to use "suggests" and "initiation".

Introduction:

Comments:

Line 61: Remove "EGFR".

Response: We have removed "EGFR" from line 61 in the Introduction.

Comments:

Line 63: ".....prognostically significant." Here it misses the citation.

Response: We have added a citation for "prognostically significant" on line 63 in the Introduction.

Comments:

Line 69: Remove "rate".

Response: We have removed it.

Comments:

Line 76: Substitute "predictive" with "prognostic".

Response: We have substituted "predictive" with "prognostic" on line 95 in the Introduction.

Comments:

Line 78: Cite "Maffezzoli M, et al. External validation of a red cell-based blood prognostic score in patients with metastatic renal cell carcinoma treated with first-line immunotherapy combinations. Clin Exp Metastasis. 2024;41(2):117-129."

Response: Thank you for your suggestion. We have cited this ariticle.

Comments:

Line 92: Use "effectiveness" than "efficacy".

Line 95: Substitute "predictive" with "prognostic".

Response: These revisions have been made in the introduction.

Methods:

Comment: Line 139: Introduce "NSCLC" as abbreviation.

Response: "NSCLC" has been introduced as an abbreviation.

Comments:

Line 150: Better to use "fewer times" than "less".

Response: We have changed "less" to "fewer times" on line 150 in the Statistical Analysis section.

Comments:

Statistical Analysis: the statistical plan of the study is solid and appropriate. The dual validation approach, logistic regression, ROC curve and correlation analyses, as well as the use of CDW, enhance the robustness and generalizability of the findings. However, there are several areas where the methodology could be strengthened:

1) I think that incorporating time-to-event analysis (Cox proportional hazards models) could provide additional insights about the biomarkers and their association with PFS.

Line 150: Incorporating time-to-event analysis (Cox proportional hazards models) could provide additional insights.

Response: We appreciate the positive feedback regarding our statistical plan and the robustness of our methodology. Regarding your suggestion to incorporate time-to-event analysis using Cox proportional hazards models, we acknowledge the value of this approach. However, as described in the limitations section of our manuscript, the Clinical Data Warehouse (CDW) utilized in our study does not provide information on whether an event (progressive disease, PD, in this case) occurred or not. Consequently, conducting a Cox regression analysis was not feasible. We have now revised the relevant section of our manuscript to clarify this point further:

"Logistic regression identified factors predicting ≤2 or ≥5 pembrolizumab doses as proxies for progression-free survival (PFS), and Receiver Operating Characteristic analysis was used to examine their predictive ability."

2) I think that exploring additional novel biomarkers or combining multiple markers into a composite score might improve predictive accuracy.

Response: In this study, we examined all possible candidate biomarkers available from the Data Warehouse (DWH). However, RDW was the only factor that remained independent and significant, even in multivariate analysis. This finding underscores the robustness of RDW as a prognostic biomarker within the constraints of the data available to us.

Comments:

Table 1: Please, clarify whether the age values are reported as median or mean, and explain ranges in brackets.

Response: We have clarified in Table 1 that age values are reported as median and explained the ranges in brackets.

Comments:

Line 181, Exploration Part: Please, specify that there's no differences in the DISTRIBUTION of age, gender, etc. between the two groups.

Response: We have revised the manuscript to specify that there were no significant differences in the distribution of age, gender, or the department in which the ICI was administered between the two groups. The added sentence is: "There were no significant differences in age, gender, or the department in which the ICI was administered in the two groups."

Comments:

Line 195: blood urea nitrogen (BUN).

Line 196: Chlorine (Cl)

Response: We have added the full forms for BUN and Cl on lines 195 and 196, respectively.

Comments:

Line 203: The reasons for selecting specific variables for the multivariate model should be clearly justified.

Response: We have revised the manuscript to clarify the selection process for the variables in the multivariate model. The added sentence is: "Of these 15 items, 5,005 models were created with 9 items as explanatory variables, and the explanatory variables of the model with the best classification performance were selected." in line 235 of revised manuscript.

Comments:

Line 206: The multivariate logistic regression includes multiple predictors, but only RDW remained significant. It might be beneficial to explore interactions between variables and other potential confounders.

Response: Among the variables used in the multivariate analysis in this study, Hb and Hct and WBC and neutrophil count have strong correlations with each other and may be multicollinear. However, there is no item with a strong correlation for RDW, and since the subsequent validation also found an association with prognosis, we do not believe that multicollinearity has a significant impact on the present results.

Comments:

Line 208: Justify the selection of the RDW threshold (15.5) with more detail.

Response: We have revised the manuscript to provide a detailed justification for the selection of the RDW threshold. The RDW threshold value of 15.5 was obtained using the Youden Index, which is described in the Methods section. This method was used to determine the optimal cutoff point for distinguishing between early discontinuation and sustained treatment groups based on RDW values.

Revised Sentence in Manuscript:

"For this model, the only variable that was significant was RDW (p=0.0008). In the ROC analysis using sustained-treatment and early-discontinuation with RDW, the Area Under the Curve was 0.60, and at an RDW value of 15.5, the sensitivity for detecting the early-discontinuation administration group was 0.41 and the specificity was 0.79 (Fig 2). This value was obtained using the Youden Index (described in Methods)."

Comment:

Validation part 2: It might be beneficial to explore interactions between variables and other potential confounders.

Response: Since this validation part only examined the validity of the factors identified in the exploratory part as prognostic predictors of RDW, other factors were not examined.

Comment:

Line 301: Use "NSCLC".

Response: "NSCLC" has been used accordingly.

Comment:

Line 318: Cite "Maffezzoli M, et al. External validation of a red cell-based blood prognostic score in patients with metastatic renal cell carcinoma treated with first-line immunotherapy combinations. Clin Exp Metastasis. 2024;41(2):117-129."

Response: Thank you for your suggestion. This citation has been added.

Discussion:

Comment: External validation using data from different institutions or a prospective cohort would strengthen the findings' generalizability.

Response: We acknowledge the importance of external validation for strengthening the generalizability of our findings. In the revised manuscript, we have addressed this point as follows: "These limitations could be addressed by external validation data sets or validation with a prospective cohort, which would strengthen the generalizability of the findings."

Reviewer #2 Comments:

Comments:

• Abstract

Line 36: Expand EMR when using for the first time.

Response: The term "Electronic Medical Record (EMR)" has been expanded at its first occurrence in the manuscript.

Comments:

Line 36: Expand NSCLC when using for the first time.

Response: The term "Non-Small Cell Lung Cancer (NSCLC)" has been expanded at its first occurrence in the manuscript.

Comments:

Clarify the methodology in the abstract: Were there two cohorts? Was the study only done on NSCLC patients? Mention the number of patients in each cohort (n=__). Line 41: "... utilized CDW for discovery and EMR/CDW for validating prognostic biomarkers for ICI treatment using the number of doses of ICI as a proxy." Or restructure the abstract to make it clear that the question is about finding biomarkers to predict ICI response, with an intermediate step being finding a PFS proxy.

Response 1: The methodology in the abstract has been clarified to specify the cohorts and the patient numbers. The revised abstract reads:

"This retrospective study utilized a CDW to explore factors associated with pembrolizumab treatment duration validated in non-small cell lung cancer (NSCLC) patient cohorts from electronic medical records (EMR) and CDW. The CDW contained anonymized data on demographics, diagnoses, medications, and tests for cancer patients treated with ICIs between 2017-2022. Logistic regression identified factors predicting ≤2 or ≥5 pembrolizumab doses as proxies for progression-free survival (PFS), and Receiver Operating Characteristic analysis was used to examine their predictive ability. These factors were validated by correlating doses with PFS in the EMR cohort and re-testing their significance in the CDW cohort with other ICIs. This dual approach utilized the CDW for discovery and EMR/CDW cohorts for validating prognostic biomarkers before ICI treatment."

Response 2: The number of patients in each cohort has been mentioned: "A total of 609 cases (428 in the exploratory cohort and 181 in the validation cohort) from CDW and 44 cases from EMR were selected for study."

Comments:

• Introduction

Line 63: Provide a citation for immune-related adverse events. Explain the tail effect and early non-response.

Response: We have revised the manuscript to include a citation for immune-related adverse events and to explain the tail effect and early non-response. The revised sentence reads: "Compared to traditional cytotoxic chemotherapeutics and molecular targeted therapies like tyrosine kinase inhibitors, ICIs have distinctive features, including long-lasting anti-tumor effects (a long tail effect), cancer progression early after initiation of treatment (early non-response)[4], and the occurrence of adverse events similar to autoimmune diseases mediated by the immune system[5], some of which can be severe and prognostically significant."

Comments:

Line 71: Clarify "various measurement methods".

Response: We appreciate the reviewer's comment on clarifying the "various measurement methods." We have revised this section to more accurately reflect our intended meaning. In the revised manuscript:

"Biomarkers such as tumor PD-L1 expression rate, microsatellite instability, and Tumor Mutation Burden are currently used to predict the effects of ICIs. However, these biomarkers have issues like insufficient predictive accuracy, variability in assessment methods (including different antibodies used for PD-L1 evaluation and potential inter-observer variability), and heterogeneity within the same tumor[7, 8]. Therefore, there is a demand for the development of biomarkers that can more accurately predict the therapeutic effects of ICIs."

Methodology:

Comments:

State why the authors selected only lung cancer patients for the validation cohorts.

Response: In the exploratory part of the study, we analyzed all patients who received ICI regardless of the type of cancer in order to ensure the number of cases. However, the prognosis of each cancer type is different, and it is desirable to analyze each type of cancer separately. Therefore, in the exploratory part, potential prognostic factors were identified, and in the validation part, whether the factors obtained in the exploratory part had the same predictive ability for a single cancer type was verified.

Comments:

Explain how the authors selected the number of doses for early-discontinuation and sustained-treatment.

Response: We have revised the manuscript to clarify the selection of the number of doses for early-discontinuation and sustained-treatment. The early-discontinuation group was defined as receiving two or fewer doses, and the sustained-treatment group was defined as receiving five or more doses. This threshold was selected by reference to the overall distribution and to ensure a sufficient number of cases for statistical analysis. Cases where the period between the first dose and the last day of CDW storage was less than 12 weeks were excluded as it was impossible to determine whether they belonged to the sustained-treatment group or not. This is detailed in the revised manuscript.

Revised Section in Manuscript:

"Early-discontinuation group was defined as two or fewer doses and sustained-treatment was defined as five or more doses. The analysis included 320 subjects. Cases in which the period between the first dose and the last day of CDW storage was less than 12 weeks were excluded because it was impossible to determine whether they belonged to the sustained-treatment group or not. There were 99 cases in the early-discontinuation group and 221 cases in the sustained-treatment group."

Comments:

Mention the statistical test used for group comparisons in the text as well as in the table captions.

Response: We have revised the manuscript to mention the statistical tests used for group comparisons in both the text and the table captions. For example, in Table 1, we stated that the p-values were calculated by comparing the short-term and long-term groups using the Mann-Whitney U test. Similarly, other tables and figures have been updated to include the relevant statistical tests used.

Revised Text and Table Example:

"Table 1: Patient Characteristics

The p-values were calculated by comparing the short-term and long-term groups. Mann-Whitney's U test was used to compare patient backgrounds in each group."

4. Results:

Comments:

Mention the criteria used to select variables for the multivariable regression.

Response: We have revised the manuscript to clarify the selection process for the variables in the multivariate model. The added sentence is: "Of these 15 items, 5,005 models were created with 9 items as explanatory variables, and the explanatory variables of the model with the best classification performance were selected." in line 235 of revised manuscript.

Comments:

State if RDW was directly compared with PFS in the first validation cohort.

Response: This validation part was intended to follow the methodology of the exploration part, so no direct comparison of RDW and PFS was made.

Comments:

Compare patient characteristics of the exploratory cohort and the validation cohorts.

Response: We appreciate the reviewer's suggestion to compare patient characteristics between the exploratory cohort and the validation cohorts. We have carefully considered this point and would like to offer the following explanation:

In our study design, the data available for comparison across all cohorts is limited primarily to age and sex. While we understand the importance of comparing cohort characteristics, we believe that a comparison based solely on these two factors may not provide meaningful insights into the cohorts' similarities or differences.

Comments:

Table 6: Perform a subgroup comparison for nivolumab, pembrolizumab, and atezolizumab (n%) between the early-discontinuation group and the sustained-treatment group instead of using an overall p-value.

Response: We appreciate the reviewer's suggestion to perform a subgroup comparison for nivolumab, pembrolizumab, and atezolizumab between the early-discontinuation group and the sustained-treatment group. However, we would like to point out that the number of patients treated with each of these drugs varies significantly in our dataset. Given this disparity, we believe that a subgroup analysis may not yield meaningful or statistically robust insights. Instead, we have presented the overall p-value to provide a more comprehensive and balanced view of the data. We hope this approach is acceptable and remains open to any further suggestions you might have for enhancing our analysis.

5. Discussion:

Comments:

It is interesting and logical that the number of pembrolizumab doses correlates with progression-free survival. Has any other study reported using the number of doses as a PFS proxy?

Response: We appreciate the reviewer's interest in our approach. As far as we could find, there were no studies using a similar approach of correlating the number of pembrolizumab doses with progression-free survival (PFS) as a proxy. Our study appears to be pioneering in this aspect, and we believe that this novel method provides valuable insights into the treatment outcomes with immune checkpoint inhibitors.

Comments:

Clarify the weak negative correlation between RDW and actual PFS.

Response: We appreciate the reviewer's comment regarding the correlation between RDW and actual PFS. In the revised manuscript, we have added a clarification in the "Results" section under the subsection "Validation part 1: Correlation Between Pembrolizumab Administration and Progression-Free Survival in Lung Cancer Patients." We have detailed the findings from our validation part, which showed a weak negative correlation between RDW values and the number of pembrolizumab administrations, with a correlation coefficient of r = -0.30 (p = 0.049).

6. Language Improvements:

Comments:

Line 37: Use "between" instead of "from."

Use active voice wherever possible. Split sentences into two instead of using long sentences (Line 60-63).

Line 300: Remove "the three ICIs."

Response: These language improvements have been made throughout the manuscript.

As for Journal Requirement, we have modified our manuscript.

1. Please ensure that your manuscript meets PLOS ONE's style requirements, including those for file naming. The PLOS ONE style templates can be found at

https://journals.plos.org/plosone/s/file?id=wjVg/PLOSOne_formatting_sample_main_body.pdf and <https://journals.plos.org/plosone/s/file?id=ba62/PLOSOne_formatting_sample_title_authors_affiliations.pdf>

Response: The revised version of our manuscript has been carefully formatted to meet PLOS ONE's style requirements. We have closely followed the style templates provided in the links you shared, ensuring compliance with both the main body formatting guidelines and the title, authors, and affiliations formatting guidelines. This includes adherence to the specified file naming conventions. We have thoroughly reviewed our manuscript to ensure it aligns with all aspects of PLOS ONE's style requirements.

2. In the online submission form, you indicated that "Data cannot be shared publicly due to ethical considerations. The data underlying the results presented in this study are available from the Yokohama University Ethics Committee. Interested researchers who meet the criteria for access to confidential data can request the data by contacting the corresponding author."

All PLOS journals now require all data underlying the findings described in their manuscript to be freely available to other researchers, either 1. In a public repository, 2. Within the manuscript itself, or 3. Uploaded as supplementary information.

This policy applies to all data except where public deposition would breach compliance with the protocol approved by your research ethics board. If your data cannot be made publicly available for ethical or legal reasons (e.g., public availability would compromise patient privacy), please explain your reasons on resubmission and your exemption request will be escalated for approval.

Response: We have thoroughly reviewed our reference list and can confirm that it is complete and correct. We have not cited any retracted papers in our manuscript. All references used are current and relevant to our study.

3. Your ethics statement should only appear in the Methods section of your manuscript. If your ethics statement is written in any section besides the Methods, please move it to the Methods section and delete it from any other section. Please ensure that your ethics statement is included in your manuscript, as the ethics statement entered into the online submission form will not be published alongside your manuscript.

Response: The ethics statement in our manuscript was already appropriately placed in the Methods section in the first version we submitted. We have double-checked to confirm that it appears only in the Methods section and is not duplicated elsewhere in the document.

4. Please review your reference list to ensure that it is complete and correct. If you have cited papers that have been retracted, please include the rationale for doing so in the manuscript text, or remove these references and replace them with relevant current references. Any changes to the reference list should be mentioned in the rebuttal letter that accompanies your revised manuscript. If you need to cite a retracted article, indicate the article’s retracted status in the References list and also include a citation and full reference for the retraction notice.

Response: We have thoroughly reviewed our reference list and can confirm that it is complete and correct. We have not cited any retracted papers in our manuscript. All references used are current and relevant to our study. No changes to the reference list were necessary, as we have ensured the accuracy and appropriateness of all citations prior to submission.

We hope that the revised manuscript now meets the journal’s requirements. We have uploaded the revised manuscript with tracked changes, a clean version of the revised manuscript, and this response letter. Thank you again for your valuable feedback and consideration.
